# Histone Deacetylase (HDAC) Gene Family in Allotetraploid Cotton and Its Diploid Progenitors: In Silico Identification, Molecular Characterization, and Gene Expression Analysis under Multiple Abiotic Stresses, DNA Damage and Phytohormone Treatments

**DOI:** 10.3390/ijms21010321

**Published:** 2020-01-03

**Authors:** Muhammad Imran, Sarfraz Shafiq, Muhammad Kashif Naeem, Emilie Widemann, Muhammad Zeeshan Munir, Kevin B. Jensen, Richard R.-C. Wang

**Affiliations:** 1 School of Life Sciences, Tsinghua University, Beijing 100084, China; imran_m1303@yahoo.com; 2 State Key Laboratory of Plant Cell and Chromosome Engineering, Institute of Genetics and Developmental Biology, Chinese Academy of Sciences, Beijing 100101, China; kashifuaar102@hotmail.com; 3 Department of Environmental Sciences, COMSATS University Islamabad, Abbottabad Campus, Abbottabad 22060, Pakistan; 4 Department of Biology, University of Western Ontario, 1151 Richmond St, London, ON N6A5B8, Canada; ewidema4@uwo.ca; 5 Beijing Advanced Innovation Center for Tree Breeding by Molecular Design, National Engineering Laboratory for Tree Breeding, Key Laboratory of Genetics and Breeding in Forest Trees and Ornamental Plants, College of Biological Sciences and Technology, Beijing Forestry University, Beijing 100083, China; zeeshanmunir1270@gmail.com; 6 Forage & Range Research, United States Department of Agriculture, Agricultural Research Service, Logan, UT 84322, USA; kevin.jensen@usda.gov

**Keywords:** histone deacetylases, genome-wide analysis, fiber, abiotic stress, cotton

## Abstract

Histone deacetylases (HDACs) play a significant role in a plant’s development and response to various environmental stimuli by regulating the gene transcription. However, *HDACs* remain unidentified in cotton. In this study, a total of 29 HDACs were identified in allotetraploid *Gossypium hirsutum*, while 15 and 13 HDACs were identified in *Gossypium arboretum* and *Gossypium raimondii*, respectively. *Gossypium* HDACs were classified into three groups (reduced potassium dependency 3 (RPD3)/HDA1, HD2-like, and Sir2-like (SRT) based on their sequences, and *Gossypium* HDACs within each subgroup shared a similar gene structure, conserved catalytic domains and motifs. Further analysis revealed that *Gossypium* HDACs were under a strong purifying selection and were unevenly distributed on their chromosomes. Gene expression data revealed that *G. hirsutum* *HDACs* were differentially expressed in various vegetative and reproductive tissues, as well as at different developmental stages of cotton fiber. Furthermore, some *G. hirsutum HDACs* were co-localized with quantitative trait loci (QTLs) and single-nucleotide polymorphism (SNPs) of fiber-related traits, indicating their function in fiber-related traits. We also showed that *G. hirsutum HDACs* were differentially regulated in response to plant hormones (abscisic acid (ABA) and auxin), DNA damage agent (methyl methanesulfonate (MMS)), and abiotic stresses (cold, salt, heavy metals and drought), indicating the functional diversity and specification of HDACs in response to developmental and environmental cues. In brief, our results provide fundamental information regarding *G.* *hirsutum* *HDACs* and highlight their potential functions in cotton growth, fiber development and stress adaptations, which will be helpful for devising innovative strategies for the improvement of cotton fiber and stress tolerance.

## 1. Introduction

Cotton is the most important crop worldwide that has contributed a lot for the textile industry through its natural and renewable fiber. *Gossypium hirsutum* is a principal source of commercial cotton production [1], and thus holds a significant value in the world economy. *G. hirsutum*, a natural allotetraploid (AADD, 2*n* = 4*x* = 52), originated approximately 1–2 million years ago from the interspecific hybridization between the diploid A genome *Gossypium arboretum* (AA, 2*n* = 2*x* = 26) and D genome *Gossypium raimondii* (DD, 2*n* = 2*x* = 26) [2,3]. In addition to its economic value, cotton is a model system for plant polyploidy, cell elongation and cell wall biosynthesis [4,5,6]. A fluctuating climate adversely affects cotton yield and fiber quality, and thus motivates us to explore the strategies that can combat environmental stresses and produce high quality fiber [7,8]. The available genomic data on *G. hirsutum* [9,10], *G. arboretum* [11] and *G. raimondii* [12] provide an excellent platform to identify the new candidate genes that can confer resistance to biotic and abiotic stresses, and produce high-quality fiber. Attempts have been made to identify different genes; however, the identification of epigenetic regulators in cotton and their involvement in fiber development and stress adaptation is very limited.

Plants use several epigenetic modifications, including post-transcriptional histone modifications and DNA methylation, to tightly govern the gene expression and phenotypic plasticity [13,14,15]. Histones are subjected to numerous covalent modifications, including acetylation, methylation, ubiquitination, and sumoylation, which impact the gene expression by recruiting the histone writers, erasers and readers [14]. Histone acetylation is the addition of an acetyl group to the N-terminal of histones, and is a mark of transcriptional activation in eukaryotes. Histone acetylation has been reported in different lysine residues of histones, e.g., H3 (K4, K9, K14, K18, K23, K27), H4 (K5, K8, K12, K16) and H2B (K5, K12, K15, K20) [16]. Histone deacetylases (HDACs) maintain the homeotic balance of histone acetylation by removing the acetyl group and are linked to transcriptional repression and gene silencing. HDACs are well conserved in different organisms, including human, yeast and plants, and are generally classified into three distinct groups in plants; the reduced potassium dependency 3 (RPD3/HDA1) superfamily, the HD2-like family, and the silent information regulator 2 (SIR2) family [17]. HD2-like family is plant specific. The number of HDAC genes are variable among different plants. For example; *Arabidopsis thaliana* [18] and rice (*Oryza sativa*) [19] contain 18 HDACs, soybean (*Glycine max*) contains 24 HDACs [20], *Vitis vinifera* contains 13 [21], litchi (*Litchi chinensis* Sonn. cv. Feizixiao) contains 11 [22], while maize (*Zea mays*) and tomato (*Solanum lycopersicum*) contain 15 HDACs [23,24].

In *Arabidopsis*, HDACs play an important role in the response to different stresses, as well as in plant development [18,25,26,27]. *Arabidopsis* HDA6 is involved in drought stress tolerance [28] and regulates the jasmonate-associated stress response [29,30]. The *Athda9* mutant showed enhanced tolerance to salt and drought stress, indicating that HDA9 may act as a negative repressor of the plant’s stress response [31,32,33]. Overexpression of *HD2D*/*C* in *Arabidopsis* showed an increased tolerance to drought and salt stress [34,35]. In rice, overexpression of *OsHDT701* also resulted in increased tolerance to drought and salt stress [36,37]. In contrast, overexpression of *OsHDA705* in rice showed a decreased salt and abscisic acid (ABA) stress tolerance during seed germination [38]. After a short-day treatment, the transcriptional dynamics of the leaf and shoot apical meristem were compared in soybean [20], and the results showed that the expression of several *HDACs* was altered during the floral initiation process. For example; the expression of HD2 members *Glyma12g30600.1* and *Glyma12g09000.1* was decreased with a short-day treatment. In tomato, SlHDA1, SlHDA3 and SlHDA4, belonging to the RPD3/HDA1 family, were shown to interact with tomato agamous 1 (TAG1) and tomato MADS BOX 29 (TM29), which are associated with reproductive development [23]. These studies indicate the diversity and specificity of HDACs in plant stress response and development. Interestingly, HDACs from the HD2-like family interacts with the RPD3/HDA1 family members [39], suggesting that multiple HDACs participate in multi-protein complexes to regulate the gene expression. HDACs also interact with other epigenetics regulators, suggesting HDACs may act in combination with other chromatin regulators to landscape the transcription. For example, HDA6 interacts with DNA methyltransferase MET1 [40], histone demethylase Flowering Locus D (FLD) [41], and histone methyltransferases SUPPRESSOR OF VARIEGATION 3-9 HOMOLOG 4/5/6 (SUVH4/5/6) [27]. These studies highlight the importance of HDACs in gene expression regulation during plant development and in response to environmental stimuli. However, the identification and function of HDACs in cotton is yet to be investigated.

Here, we first identified *HDACs* from *G. hirsutum*, *G. arboretum* and *G. raimondii*, and then comprehensively analyzed them through the phylogenetic classification, gene structure and chromosomal location, domain organization, and the *cis-*regulatory elements in their promoters. In addition to these bioinformatics analyses, the gene expression patterns of *G. hirsutum HDACs* were studied during the different stages of fiber development, phytohormone treatment, and diverse abiotic stresses, including Zn and Cd metal stresses, cold, drought and salt. Thus, our comprehensive analysis provides a fundamental understanding of *Gossypium* HDACs in cotton growth, development and stress responses. This study will lead to a long-term improvement of cotton and will be useful for functional genomic studies on the regulation of histone acetylation in cotton.

## 2. Results

### 2.1. Identification and Phylogenetic Analysis of Gossypium HDAC

To identify the HDACs in the genomes of *G. hirsutum*, *G. raimondii* and *G. arboretum*, *Arabidopsis* query sequences were used to perform a systematic blast search. After verification using Pfam and InterProScan databases, a total of 29 *G. hirsutum* HDACs (GhHDACs), 15 *G. arboretum* HDACs (GaHDACs), and 13 *G. raimondii* HDACs (GrHDACs) were identified (Table S1). ExPASy was further used to analyze the properties of the identified HDACs. We found that the open reading frame (ORF) length of *Gossypium* HDACs ranged from 606–2004 bp, which encoded the polypeptides from 201–667 amino acids. In addition, the predicted molecular weight of *Gossypium* HDACs ranged from 21–75 kDa. In addition, the calculated p*I* values ranged from 4.32–8.96 (Table S1).

**Figure 1:**
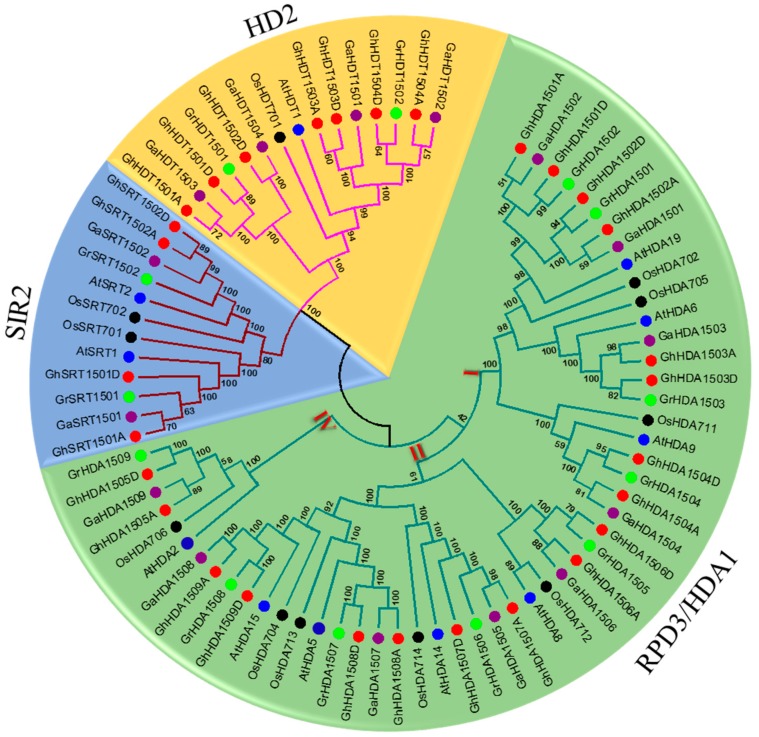
Phylogenetic relationships of histone deacetylases (HDACs) from *G. hirsutum*, *G. raimondii*, *G. arboretum*, *Arabidopsis thaliana* and *Oryza sativa*. The unrooted phylogenetic tree was constructed with MEGA 6 using the neighbor-joining method and the bootstrap analysis was performed with 1000 replicates. The HDACs from *G. hirsutum*, *G. raimondii*, *G. arboretum*, *Arabidopsis thaliana* and *Oryza sativa* are marked with red, green, purple, blue and black dots, respectively. The branches of each subgroup are indicated in a specific color. RPD3: reduced potassium dependency 3, SIR2: silent information regulator 2.

To determine the evolutionary relationship among the newly identified *Gossypium* HDACs (*G. raimondii*, *G. arboretum* and *G. hirsutum*), as well as with other species, an unrooted phylogenetic tree using MEGA 6.0 was generated. We used previously identified *Arabidopsis* [18] and rice [19] HDACs, in addition to newly identified 57 *Gossypium* HDACs, to generate the phylogenetic tree using the neighbor-joining (NJ) method (Figure 1). The phylogenetic results showed that *Gossypium* HDACs could be classified into three distinct classes (i.e., RPD3/HDA1, HD2-like, and Sir2-like (SRT), similar to *Arabidopsis* and rice. In addition, the RPD3/HDA1 class could also be divided into three distinct subgroups, class I for the RPD3 group, class II for the HDA1-like group, and class IV for the HDA2 group. We further found that the RPD3/HDA1, HD2-like, and SRT groups comprised both mono and dicot species, and the total number of genes in RPD3/HDA1 (52) was greater than the number of genes in the HD2-like (15) and SRT (12) subgroups. We also observed that the number of HDACs from each species was different within each group and *Gossypium HDACs* clustered closely to *Arabidopsis* in contrast with rice, indicating their close phylogenetic relationship with *Arabidopsis*. Nevertheless, their gene numbers were not similar within the group.

### 2.2. Phylogenetic Classification, Gene Structure, and Conserved Domain Analysis of Gossypium HDAC Genes

**Figure 2:**
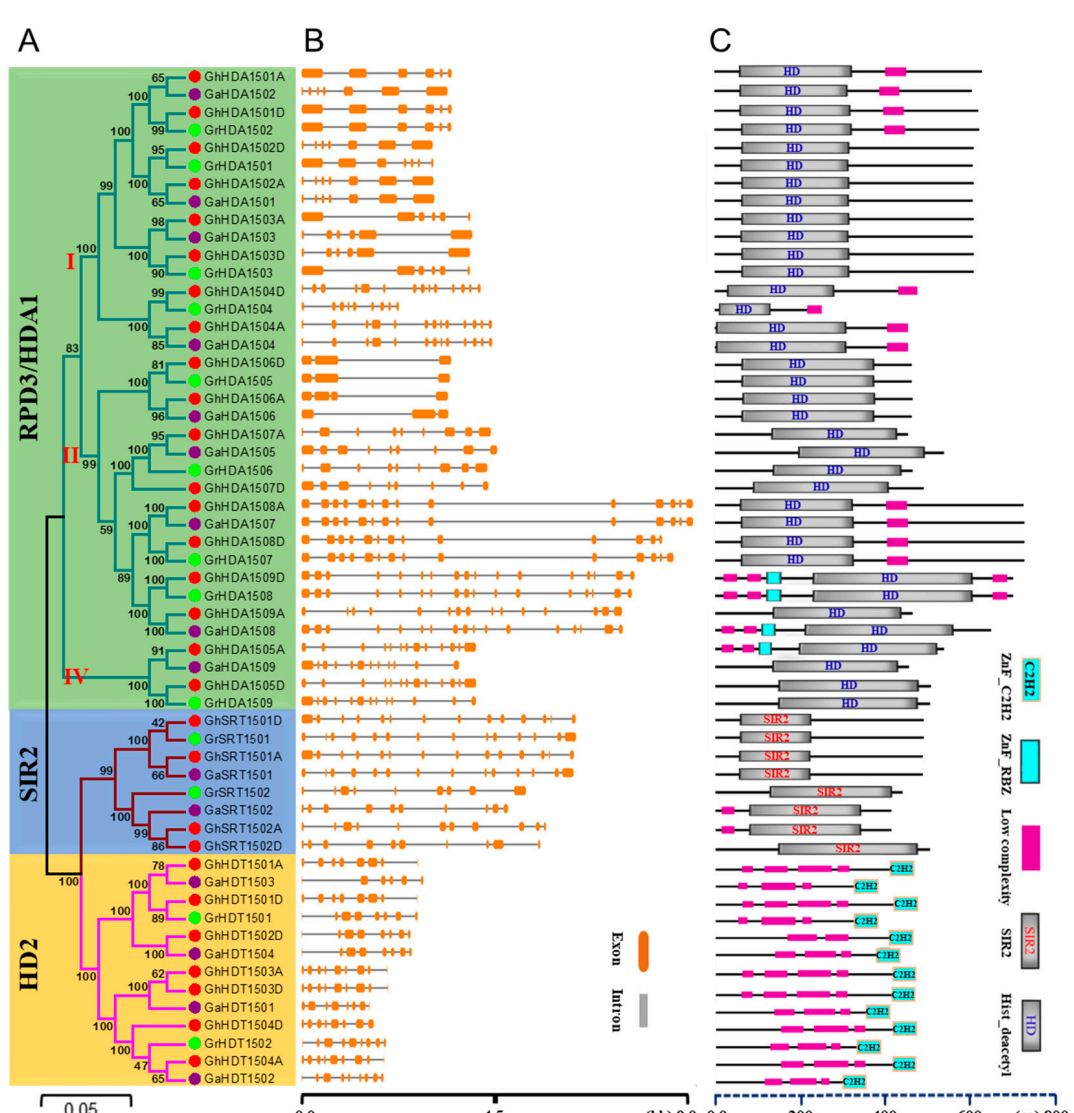
Phylogenetic relationships, gene structure analysis, and domain organization of HDACs in *G. hirsutum*, *G. raimondii* and *G. arboretum*. (**A**) The unrooted phylogenetic tree was constructed with MEGA 6 using the neighbor-joining method and the bootstrap analysis was performed with 1000 replicates. The *HDACs* from *G. hirsutum*, *G. raimondii*, and *G. arboretum* are marked with red, green, and purple dots, respectively. The branches of each subgroup are indicated in a specific color. (**B**) The intron-exon structure of *HDACs* from *G. hirsutum*, *G. raimondii* and *G. arboretum*. Orange boxes and grey lines represent exons and introns, respectively. (**C**) Domain organization of HDACs from *G. hirsutum*, *G. raimondii* and *G. arboretum*.

In order to confirm the subgroup classification and to determine the evolutionary relationship among the *Gossypium* HDACs, we generated another phylogenetic tree using the NJ method (Figure 2A). This phylogenetic analysis further confirmed the three distinct groups RPD3/HDA1, HD2-like, and SRT, as well as three subgroups of RPD3/HDA1 class in *Gossypium* HDACs. In *G. raimondii* and *G. arboretum*, SRT and RPD3/HDA1 groups contained two and nine members, respectively. However, *G. arboretum* contained four members of HD2-like, while *G. raimondii* contained only two members of HD2-like. Moreover, in *G. hirsutum*, RPD3/HDA1, SRT and HD2-like classes contained 18, 4, and 7 members, respectively. In *G. hirsutum*, the subgroups I, II and IV of RPD3/HDA1 contained eight, eight, and two members, respectively. In general, *G. raimondii* and *G. arboretum* had their orthologs in allotetraploid *G. hirsutum*.

We further analyzed the *HDACs* gene structure from *G. hirsutum*, *G. raimondii*, and *G. arboretum* (Figure 2B). Our results showed that RPD3/HDA1, HD2-like and SRT classes contained different numbers and lengths of exons/introns. Moreover, three distinct subgroups of RPD3/HDA1 also showed different numbers and lengths of intron/exons. For example, the number and length of intron/exons were greater in subgroup I of RPD3/HDA1 than that of HD2-like, as well as other subgroups of RPD3/HDA1. Thus, consistent with the phylogenetic analysis, the gene structure in terms of intron/exons was quite similar within the subgroup or different classes of *Gossypium HDACs*. Furthermore, the *HDACs*’ gene structure from *G. raimondii* and *G. arboretum* was largely similar to their orthologs in *G. hirsutum*.

To gain insight into the *Gossypium* HDACs’ putative functional diversification, a Multiple Expectation Maximization for Motif Elicitation (MEME) analysis was also performed and identified a total of eight conserved motifs in *Gossypium* HDACs (Figure S1). Then, we analyzed the domain organization of *Gossypium* HDACs (Figure 2C). The results showed that the RPD3/HDA1 class contained the conserved HD domain, and the SRT class contained the conserved SIR2 domain. Moreover, the HD2-like class contained the conserved C2H2 type zinc finger domain. In addition to conserved domains, some members of RPD3/HDA1 contained an additional zinc finger- Ran-binding domain (RBZ). In brief, HDACs from *G. hirsutum*, *G. raimondii* and *G. arboretum* contained a similar domain organization to that of their counterparts in *Arabidopsis* and rice.

### 2.3. Genomic Localization and Gene Duplication Analysis of Gossypium HDACs

**Figure 3:**
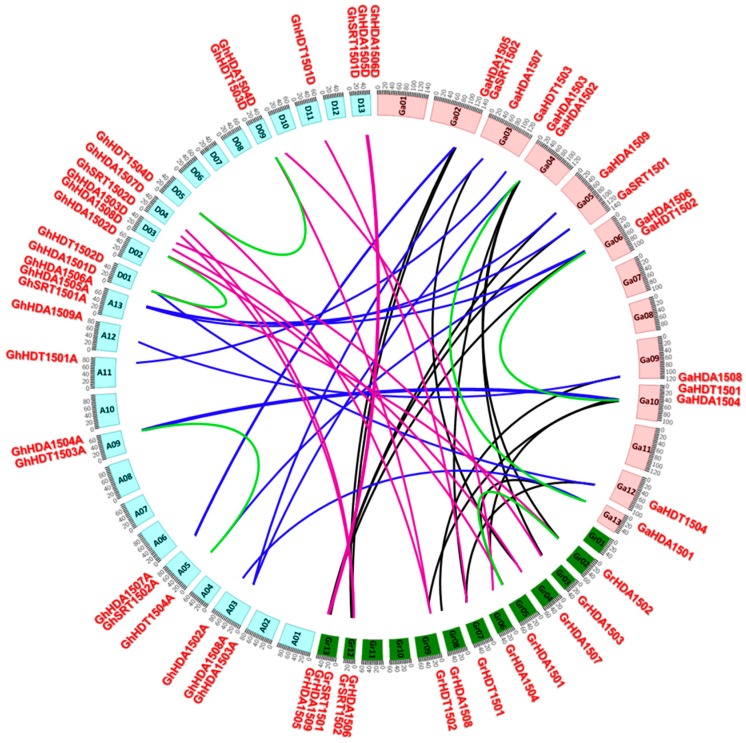
Chromosomal distribution and gene duplication of *HDACs* in *G. hirsutum*, *G. raimondii*, and *G. arboretum*. The chromosome numbers are indicated in boxes and represented as A1–A13 and D1-D13 for G*. hirsutum*, Ga1–Ga13 for *G. arboretum*, and Gr1–Gr13 for *G. raimondii*. Segment duplication of the *HDAC* genes is represented using green, while blue and purple represent the orthologous gene pairs between tetraploids and diploids, and black lines represent the orthologous genes pairs between diploid species, respectively.

*G. hirsutum HDACs* were mapped to their corresponding chromosomes (Figure 3) and the results showed that *Gossypium HDACs* were unevenly distributed on their chromosomes. For example, a total of 29 *G. hirsutum HDACs* were physically located on 15 chromosomes and their distribution on each chromosome was also uneven. The RPD3/HDA1 group of *G. hirsutum HDACs* was localized on chromosomes 1, 2, 3, 4, 9 and 13, while the HD2-like group of *G. hirsutum HDACs* was localized on chromosomes 1, 5, 9 and 11. Furthermore, the SIR2 group of *G. hirsutum HDACs* localized on chromosomes 4 and 13. We also mapped *HDACs* from *G. raimondii* and *G. arboretum* to their chromosomes. The results showed that similar to *G. hirsutum*, *G. raimondii* and *G. arboretum* also contained uneven numbers of *HDACs* on their chromosomes. However, the *HDACs* of *G. raimondii* were evenly distributed on their chromosomes, in contrast to *G. arboretum*. Furthermore, we observed that RPD3/HDA1, HD2-like and SIR2 group members of *HDACs* were localized to different chromosomes in both diploids (*G. raimondii* and *G. arboretum*), as well as in the allotetraploid (*G. hirsutum*). For example, the HD2-like group member *HDT1501* was localized on chromosome 11 in *G. hirsutum*, on chromosome 7 in *G. raimondii*, and on chromosome 10 in *G. arboretum*. We next investigated the gene duplication events in *Gossypium HDACs* and found three segmental duplications in *G. hirsutum* (*GhHDA1501D*/*GhHDA1502D*, *GhHDT1503A*/*GhHDT1504A*, *GhHDT1504D*/*GhHDT1503D*), found two segmental duplications in *G. arboretum* (*GaHDA1501*/*GaHDA1502*, *GaHDT1502*/*GaHDT1501*), and found one segmental duplication in *G. raimondii* (*GrHDA1502*/*GrHDA1501*). Furthermore, we also calculated the *Ka*/*Ks* ratio of each duplicated genes pair (Table S2), and found that the *Ka*/*Ks* ratio was less than 0.22 in all the duplicated genes of *Gossypium HDACs*. This indicates that the duplicated *Gossypium HDACs* were under strong purifying selection pressure.

### 2.4. Cis-Elements in the Promoter of G. hirsutum HDAC Genes

We next scanned the putative *cis-*elements in the promoter of *G. hirsutum HDACs* (1500 bp upstream of the transcription start site) in order to gain more insight into their function (Figure S2, Tables S3 and S4). The results revealed that *G. hirsutum HDACs* carry TATA and CAAT box core *cis*-elements, stress and development response elements, and phytohormones response elements. Although the RPD3/HDA1, HD2-like, and SRT groups shared a majority of *cis-*regulatory elements in their promoter, some of the *cis-*elements were found absent in certain groups. For example, Box-W1 (fungal elicitor responsive element), CAT box (*cis-*acting regulatory element related to meristem expression), GCN4 motif (*cis*-regulatory element involved in endosperm expression), and O2 site (*cis*-acting regulatory element involved in zein metabolism regulation) *cis*-element were absent in the SRT group of *G. hirsutum* HDACs. Similarly, the CGTCA motif (*cis*-acting regulatory element involved in MeJA-responsiveness) was only absent in the HD2-like group of *G. hirsutum* HDACs. We observed that the majority of *cis-*elements in the promoter of *HDACs* were conserved in the A and D genome of *G. hirsutum*. However, some of the *G. hirsutum HDACs* still differed in their *cis-*elements, e.g., *GhHDA1506A*/*GhHDA1506D*, highlighting the specific contribution of A or D genomes of *G. hirsutum* in regulating the particular response to different developmental and environmental cues.

### 2.5. Gene Expression Analysis

#### 2.5.1. Expression Patterns of *G. hirsutum HDACs* in Different Tissues and Under Multiple Stresses

**Figure 4:**
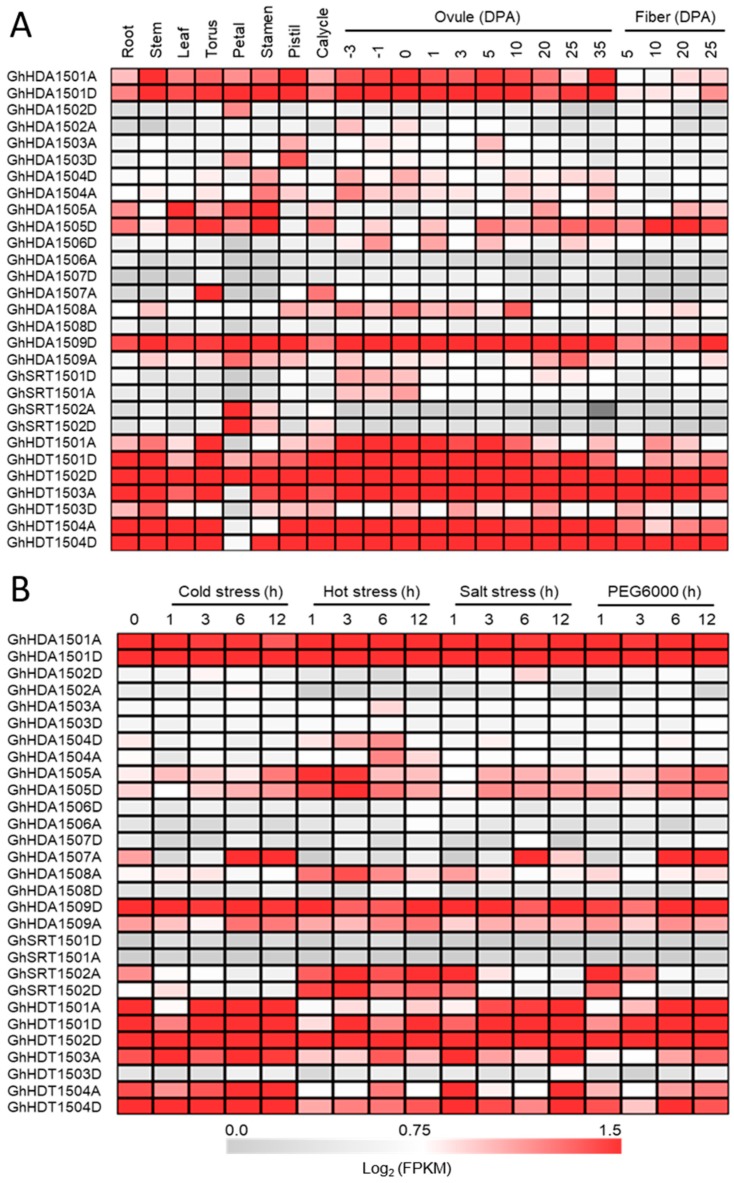
Gene expression analysis of *G. hirsutum HDACs* in different tissues and at different stages of fiber development (**A**), and in response to cold (4 °C), heat (38 °C), salt (400 mM), and PEG 6000 (20%) stress (**B**) from RNA-seq. The illumina reads of RNA-seq data were retrieved from the NCBI SRA database. The color scale on the bottom of the heatmap indicates the FPKM (Fragments Per Kilobase of transcript per Million mapped reads)-normalized log2 transformed counts. DPA: days post anthesis.

In order to unravel the possible physiological functions of *HDACs* in allotetraploid cotton, we next investigated the expression pattern of *G. hirsutum HDACs* in different tissues by analyzing the RNA-seq data (downloaded from NCBI) (Figure 4). RNA-seq analysis showed that all the *G. hirsutum HDACs* were differentially expressed in the vegetative tissues (leaf, root and stem), as well as reproductive tissues (torus, petal, stamen, pistil, calycle, and different stages of ovule development) (Figure 4A). However, some of the *G. hirsutum HDACs* were mainly expressed in reproductive tissues in contrast with vegetative tissues, e.g., *GhSRT1502A*/*D*. We also noticed that the expression of A and D genomes was also different in *G. hirsutum*, e.g., *GhHDT1503A* was highly expressed in all the vegetative and reproductive tissues in contrast with *GhHDT1503D*. These results suggest that the A and D genomes may jointly contribute for their functional role.

We next investigated the *G. hirsutum HDACs* expression at different time points of cotton cotyledon growth and root development (Figure S3). The results show that *G. hirsutum HDACs* were expressed or repressed at certain times of growth, indicating the dynamic role of *G. hirsutum HDACs* in cotton growth and development. For example, *GhHDA1503A*/*D* expression decreased with time for the seed cotyledon, while the expression of *GhHDT1501A*/*D* increased with time for root development. Because cotton encounters multiple abiotic stresses during development, we therefore analyzed the RNA-seq data of *G. hirsutum HDACs* in response to different stresses (Figure 4B). All members of the HD2-like group—*GhSRT1502*, *GhHDA1509*, *GhHDA1505* and *GhHDA1501*—showed a dynamic expression in response to cold, heat, salt and PEG 6000 stress.

#### 2.5.2. Validation of the Expression of *G. hirsutum HDACs* Genes Using qRT-PCR

##### Tissue-Specific Expression of *GhHDAC* Genes

**Figure 5:**
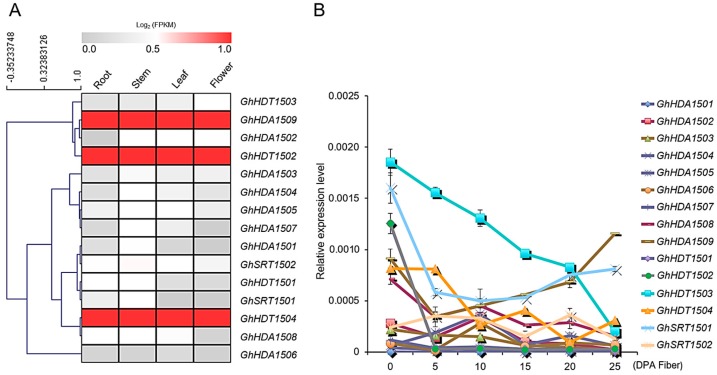
Gene expression validation of *G. hirsutum HDACs* using qRT-PCR in different tissues (**A**) and at different stages of fiber development (**B**). RNA from root, leaf, stem, flowers and different stages of fiber development were extracted and reverse transcribed. qRT-PCR was performed and the data was normalized to *UBQ7*. The relative gene expression in different tissues is presented in the heatmap. The data presented are the average of three biological replicates; the bar represents the standard deviation.

We further validated the *G. hirsutum HDACs* expression in vegetative and reproductive tissues using qRT-PCR (Figure 5A). The mRNA similarity between the A and D genomes of allotetraploid cotton was extremely high; thus, we considered *G. hirsutum GhHDAC-A* and *GhHDAC-D* as one combination (*GhHDAC*) for qRT-PCR. We found that all the 15 *G. hirsutum HDACs* showed differential expression in the analyzed tissues, similar to RNA-seq. Furthermore, the expression of *GhHDA1509* from the RPD3/HDA1 group, and *GhHDT1502* and *GhHDT1504* expression from the HD2-like group were found at the highest levels in all the tissues.

##### *GhHDACs* Expression Pattern at Different Fiber Developmental Stages

We next investigated the expression of *G. hirsutum HDACs* at different stages of fiber development (Figure 5B). Only *GhHDT1502*, *GhHDT1503*, *GhHDT1504*, *GhHDA1509* and *GhSRT1501* showed dynamic expression over the fiber development in contrast with other *G. hirsutum HDACs*. Among these five genes, the expression of HD2-like group members (*GhHDT1502*, *GhHDT1503* and *GhHDT1504*) gradually decreased with the development stages (0–25 days post anthesis (DPA)). However, the expression of *GhHDA1509* and *GhSRT1501* decreased from 0–5 DPA and then increased from 5–25 DPA of fiber development. These results indicate the functional divergence among different groups of *G. hirsutum* HDACs in fiber development.

Then, we analyzed the RNA-seq data of *G. hirsutum HDAC* genes at different stages of fiber development to separate the contribution of A and D genomes in the allotetraploid *G. hirsutum* (Figure 4A) and found that some *G. hirsutum HDACs* members belonging to D genome were preferentially expressed over those in the A genome, e.g., *GhHDA1509D* expression was higher than that of *GhHDA1509A*. Our results indicate that *G. hirsutum GhHDACs-A* and *GhHDACs-D* might jointly play an important role in fiber development.

##### Expression of *GhHDACs* in Response to Phytohormones

**Figure 6:**
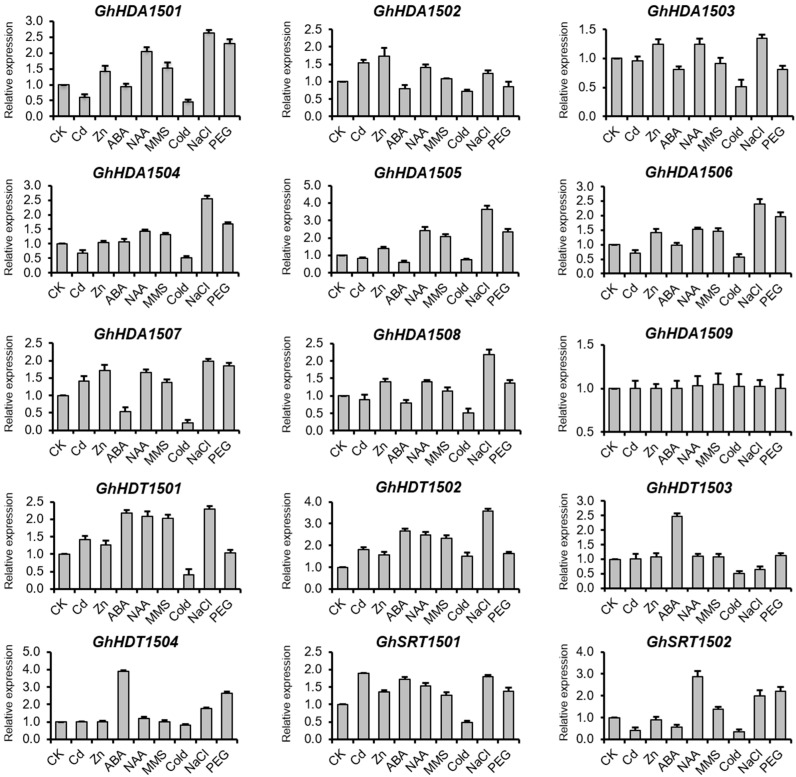
Expression pattern of *G. hirsutum HDACs* in response to abiotic stresses and phytohormones. RNA from roots after 24 h of CK (control), cadmium (Cd), zinc (Zn), abscisic acid (ABA), auxin (NAA), DNA damage (methyl methanesulfonate (MMS)), cold, salt (NaCl), and drought (PEG 4000) was extracted and reverse transcribed. *UBQ7* was used as an internal control for qRT-PCR. The data presented are the average of three biological replicates, where the bar represents the standard deviation.

Our putative *cis*-elements analysis in the promoter of *G. hirsutum HDACs* suggests that *G. hirsutum HDACs* could be regulated in response to hormones (Figure S2, Tables S3 and S4). Therefore, we investigated the expression of *G. hirsutum HDACs* in response to auxin and ABA (Figure 6). The results revealed that all *G. hirsutum HDACs* were differentially regulated in response to auxin and ABA, except *GhHDA1509*, which did not change relative to the control. In response to the ABA treatment, the expressions of *GhHDA1502*, *GhHDA1503*, *GhHDA1505*, *GhHDA1507*, *GhHDA1508* and *GhSRT1502* decreased while the expressions of *GhHDT1501*, *GhHDT1502*, *GhHDT1503*, *GhHDT1504*, and *GhSRT1501* increased relative to the control. In response to the auxin treatment, the expressions of *GhHDA1501*, *GhHDA1502*, *GhHDA1503*, *GhHDA1504*, *GhHDA1505*, *GhHDA1506*, *GhHDA1507*, *GhHDA1508*, *GhHDT1501*, *GhHDT1502*, *GhSRT1501*, and *GhSRT1502* increased, while the expressions of all other genes did not change relative to the control.

##### Expression of *GhHDACs* in Response to DNA Damage

We also investigated whether *G. hirsutum HDACs* expression was altered in response to DNA damage from methyl methanesulfonate (MMS) (Figure 6). The results showed that the expressions of *GhHDA1501*, *GhHDA1504*, *GhHDA1505*, *GhHDA1506*, *GhHDA1507*, *GhHDT1501* and *GhHDT1502* increased while the expression of other genes did not change relative to the control.

##### Expression of *GhHDACs* in Response to Abiotic Stresses

The putative *cis-*elements in the promoter suggest that *G. hirsutum HDACs* could also be regulated in response to abiotic stresses. We next investigated the expression of *G. hirsutum HDACs* in response to metal stress (Cd and Zn), salt stress (NaCl), cold, and drought stress (PEG 4000) using qRT-PCR (Figure 6). The results revealed that all *G. hirsutum HDACs* were differentially regulated in response to abiotic stresses, except *GhHDA1509*, which did not respond to any tested treatments. In response to Cd stress, the expressions of *GhHDA1501*, *GhHDA1504*, *GhHDA1505*, *GhHDA1506* and *GhSRT1502* decreased, while the expressions of *GhHDA1502*, *GhHDA1507*, *GhHDT1501*, *GhHDT1502* and *GhSRT1501* increased relative to the control. In response to Zn stress, the expressions of *GhHDA1501*, *GhHDA1502*, *GhHDA1503*, *GhHDA1506*, *GhHDA1507*, *GhHDA1508*, *GhHDT1501*, *GhHDT1502* and *GhSRT1501* increased, while the expressions of all other genes did not change relative to the control. These results indicate the specific regulation of *G. hirsutum HDACs* in response to different metal stresses. We next investigated the expression of *G. hirsutum HDACs* in response to some of the most common abiotic stresses, i.e., cold, salt, and drought. The results showed that in response to cold, the expressions of *GhHDA1501*, *GhHDA1502*, *GhHDA1503*, *GhHDA1504*, *GhHDA1505*, *GhHDA1506*, *GhHDA1507*, *GhHDA1508*, *GhHDT1501*, *GhHDT1503*, *GhSRT1501* and *GhSRT1502* decreased, while the expression of *GhHDT1502* slightly increased. In response to salt treatment, the expression of *GhHDT1503* slightly decreased, while the expressions of all other genes (except *GhHDA1509*) increased relative to the control. In response to drought stress, the expressions of *GhHDA1501*, *GhHDA1504*, *GhHDA1505*, *GhHDA1506*, *GhHDA1507*, *GhHDA1508*, *GhHDT1502*, *GhHDT1504*, *GhSRT1501* and *GhSRT1502* increased, while the expression of *GhHDA1503* slightly decreased relative to the control.

### 2.6. Co-Localization of GhHDACs with Quantitative Trait Loci and Single-Nucleotide Polymorphisms of Fiber-Related Traits

**Figure 7:**
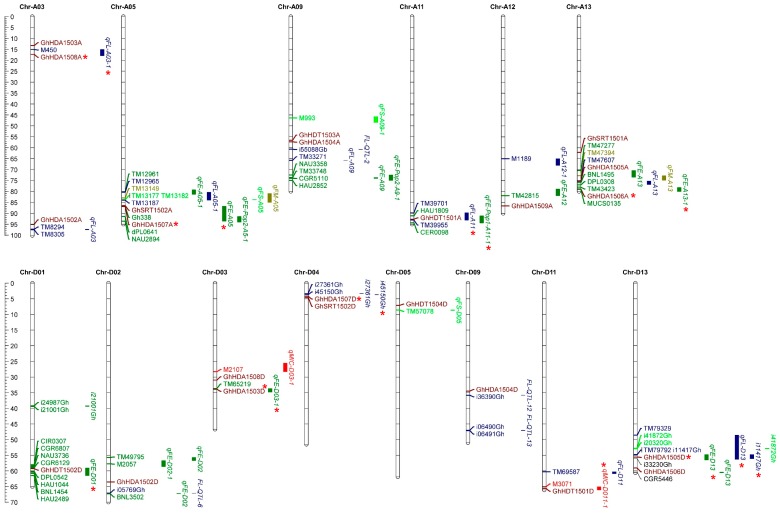
Distribution of co-localized *HDACs* on the chromosomes of the A and D genomes of *G. hirsutum*. The scale represents the physical position of genes and quantitative trait locus (QTL)-linked markers in megabases (Mb). QTLs and single-nucleotide polymorphisms (SNPs) related to fiber length (FL), fiber elongation (FE), fiber micronaire (FM), fiber strength (FS), and fiber uniformity (FU) are shown. Asterisks (*) indicate the *G. hirsutum HDACs* that were co-localized with QTLs/SNPs of different traits of fiber.

To further investigate the potential function of *HDACs* in fiber development, *G. hirsutum HDACs* were mapped to reported quantitative trait loci (QTLs) or natural variations (single-nucleotide polymorphisms (SNPs)) of fiber development, i.e., fiber length (FL), fiber elongation (FE), fiber micronaire (FM), fiber strength (FS), Micronaire (MIC), and fiber uniformity (FU) (Figure 7). From the A genome of *G. hirsutum*, only five genes were mapped inside the FE and FL QTLs: the *GhHDA1508A* gene was anchored in *qFL-A03-1* on chromosome A-03; the *GhHDA1507A* gene resided within *qFE-A05* on chromosome A-05; the *GhHDT1501A* gene was mapped inside the two QTLs, i.e., *qFL-A11* and *qFE-Pop1-A11-1* on chromosome A-11; while two genes, *GhHDA1505A* and *GhHDA1506A*, were mapped inside the QTLs *qFL-A13* and *qFE-A13-1*, respectively. However, few HDACs from the A genome of *G. hirsutum* were localized in the proximity of different fiber development QTLs. This indicates that the A genome *HDACs* of *G. hirsutum* that co-localizes inside QTLs/SNPs may directly play an important role in fiber-related traits.

From the D genome of *G. hirsutum*, five genes were anchored inside the QTLs on five chromosomes: the *GhHDT1502D* gene was mapped inside the *qFE-D01* on chromosome D-01; the *GhHDA1503D* gene was anchored in the *qFE-D03-1* on chromosome D-03; *GhHDA1507D* was presented in the Linkage Disequilibrium (LD) region of the FL natural variation (*i45150Gh*) on chromosome D-04; *GhHDT1501D* resided in the MIC-QTL (*qMIC-D011-1*) on chromosome D-11; and *GhHDA1505D* was mapped to the three QTLs, i.e., *qFE-D13*, *qFL-D13*, and the natural variation of FL (*i11417Gh*). However, few *HDACs* from the D genome of *G. hirsutum* were localized in the proximity of fiber-related QTLs. Notably, *GhHDT1501*, *GhHDA1505* and *GhHDA1507* from both the A and D genomes of *G. hirsutum* were anchored with different fiber-related QTLs, indicating that both the A and D genomes of *G. hirsutum* jointly contributed to provide fiber-related traits. Furthermore, few *HDACs* (e.g., *GhHDA1505D)* were mapped inside the QTLs of more than one fiber trait, i.e., fiber elongation and fiber length QTLs. This indicates that *G. hirsutum HDACs* may play a comprehensive role in different traits of fiber development.

## 3. Discussion

Histone acetylation plays a key role in plant development and the response to various environmental stimuli by regulating the gene transcription [14]. However, the activity of histone acetyltransferases is balanced by the histone deacetylases, which specifically remove the acetylation to repress the transcription. However, *HDACs* have not been investigated yet and compared within three genomes of cotton. In this study, we revealed that *Gossypium* HDACs could be classified into three major subgroups; e.g., RPD3/HDA1, HD2-like, and SRT, similar to *Arabidopsis* and rice. Moreover, *Gossypium* HDACs also carried the functional catalytic domains and other conserved domains, as well as motifs similar to their counterparts in *Arabidopsis* and rice (Figure 2C and Figure S1). For example, the RPD3/HDA1 class contained the conserved HD domain, the SRT class contained the conserved the SIR2 domain, and the HD2-like class contained the conserved C2H2 type zinc finger domain (Figure 2C). Furthermore, *Gossypium HDACs* clustered closely to *Arabidopsis* (Figure 1). These observations indicate that *Gossypium* HDACs could be bona fide histone deacetylases similar to their *Arabidopsis* counterparts.

Tandem and segmental duplications contribute in generating new gene subfamilies in the evolution of genome and genetic systems [42]. *G. hirsutum* contained three segmental duplications (*GhHDA1501D*/*GhHDA1502D*, *GhHDT1503A*/*GhHDT1504A*, and *GhHDT1504D*/*GhHDT1503D*). Similarly, *G. arboretum* contained two segmental duplications (*GaHDA1501*/*GaHDA1502* and *GaHDT1502*/*GaHDT1501*), and *G. raimondii* contained one segmental duplication (*GrHDA1502*/*GrHDA1501*) (Figure 3 and Table S2). Moreover, the *Ka*/*Ks* ratio suggests that duplicated genes did not diverge much during evolution (Table S2) and were likely to have functional conservation. This observation was partially validated by the overlapping expression of *GhHDT1503* and *GhHDT1504* in response to ABA treatments (Figure 6). Both *GhHDT1503* and *GhHDT1504* showed the strongest response to ABA treatment compared with all other tested treatments. However, the expression of *GhHDT1504* increased in response to NaCl and PEG relative to the control, while the expression of *GhHDT1503* decreased in response to NaCl (Figure 6). This indicates that these duplicated genes may have functional divergence in response to particular stimuli.

*G. hirsutum* is a principal source of commercial cotton production [1], and thus holds a significant value in the world economy. Interestingly, we observed that the expression of *GhHDT1502*, *GhHDT1503*, and *GhHDT1504* gradually decreased with the different stages of cotton fiber development (0–25 DPA), suggesting the dynamic levels of histone acetylation mediated by *G. hirsutum* HDACs in cotton fiber development. Fiber development is also regulated by phytohormones, including abscisic acid, auxin, gibberellic acid, ethylene, and jasmonic acid [5,43,44,45,46]. Phytohormones have been reported to alter the chromatin structure through the regulation of chromatin modifiers [47], suggesting a potential crosstalk between phytohormones and histone acetylation. Indeed, the exogenous application of auxin has been shown to decrease the H3K9ac and H3K4ac levels at the *KRP7* locus [48], while they have been shown to increase at the *SKP2B* promoter in *Arabidopsis* [49]. Our qRT-PCR results showed that ABA and auxin regulated the expression of some *G. hirsutum HDACs* relative to the control (Figure 6), indicating a change in histone acetylation levels in response to hormone treatment. Furthermore, these results also indicate the functional divergence and specific regulation of *G. hirsutum HDACs* in response to a particular phytohormone. For example, the expression of *GhHDT1502*, *GhHDT1503* and *GhHDT1504* was induced by the ABA treatment, while only the expression of *GhHDT1503* was induced by the auxin treatment (Figure 6). This specific gene expression regulation of *G. hirsutum* HDACs could also be observed at the level of RPD3/HDA1, HD2-like, and SRT group members in response to phytohormones. For example, the expression of all the members of the HD2-like group (*GhHDT1501, GhHDT1502, GhHDT1503* and *GhHDT1503*) and *GhSRT1501* from the SRT group were increased in response to ABA relative to the control, while the expression of RPD3/HDA1 group did not change relative to the control (Figure 6). However, the chromatin modifiers may also regulate hormone signaling and pathways [47]. It has been shown that HDAC inhibitors repress the auxin distribution and accumulation in the root apex by promoting the PIN-FORMED 1 (PIN1) protein degradation [50,51], indicating a crosstalk between phytohormones and HDACs in plants. Together, our results highlight that *G. hirsutum* HDACs may play an important role in fiber development via phytohormones’ dependent and/or independent pathways. Further studies are required to investigate the crosstalk of HDACs with phytohormones in cotton and the underlying epigenetic mechanism of fiber development with respect to histone deacetylation.

Double-strand break (DSB)-inducing agents have been reported to alter the histone acetylation and methylation levels in mammals [52]. Furthermore, Sirtuin 6 (SIRT6), a human deacetylase, is one of the first factors recruited to double-strand breaks (DSBs), which later recruits a chromatin remodeler Sucrose Nonfermenting 2 Homolog (SNF2H) at the DSBs in order to ensure the proper functioning of the DNA damage repair machinery [53]. These results indicate the key role of HDACs in DNA damage and repair pathways. Our qRT-PCR results showed that the expression of *GhHDA1501*, *GhHDA1504*, *GhHDA1505*, *GhHDA1506*, *GhHDA1507*, *GhHDT1501* and *GhHDT1502* was increased relative to the control in response to MMS (Figure 6), indicating that these seven *G. hirsutum HDACs* may play an important role in DNA damage repair in cotton. However, further studies are required to investigate whether and how *G. hirsutum HDACs* are involved in DNA damage and repair pathways.

Histone deacetylases have been shown to participate in different abiotic stresses, including cold, salt, and drought [33,34,36,54]. For example, the *Athda9* mutant showed enhanced tolerance to salt and drought stress [31,33], while the overexpression of *HD2D*/*C* showed increased tolerance to drought and salt stress in *Arabidopsis* [34,35]. Similarly in rice, the overexpression of *OsHDT701* also resulted in increased tolerance to drought and salt stresses [36,38]. Furthermore, in maize, H3K9, H4K5 and H4K4 acetylation levels decreased in response to cold treatment, indicating a key role of HDACs in plant adaptation to stress responses [55]. Our qRT-PCR results indicated the specific and differential gene expression regulation of *G. hirsutum HDACs* in response to particular stresses (Figure 6), suggesting an important role of *G. hirsutum HDACs* in cotton’s adaptation to various abiotic stresses. Interestingly, HDACs from the HD2-like group were shown to interact with the RPD3/HDA1 family members in *Arabidopsis* [39]. Our qRT-PCR results showed that the expression of the majority of *G. hirsutum HDACs* from PRD3/HDA1, HD2-like and SRT groups was increased in response to NaCl and PEG (Figure 6), suggesting that multiple *G. hirsutum* HDACs might also work together in response to particular stimuli in cotton. *AtHDA6* interacts with DNA methyltransferases MET1 [40], histone demethylase FLD [41], and histone methyltransferases SUVH4/5/6 [27], indicating that HDACs interact with other chromatin modifiers to regulate the gene expression. However, further studies are required to investigate whether and how *G. hirsutum* HDACs cooperate with other chromatin modifiers to control the gene expression involved in developmental processes and plant adaptation to environmental stimuli.

*G. hirsutum HDACs*, such as *GhHDT1501-A* and *GhHDA1505-D*, were co-localized with different fiber-related QTLs/SNPs (Figure 7), indicating their role in different traits of cotton fiber. Moreover, it further supports our previous conclusion that both the A and D genomes of *G. hirsutum* jointly contribute to different traits of cotton fiber. Interestingly, some *G. hirsutum HDACs*, such as *GhHDT1502* that co-localized with QTL of fiber elongation, also showed strong expression in root, stem, leaves, and flower (Figure 5A). Moreover, *GhHDT1502* also showed dynamic expression during different stages of fiber elongation (Figure 5B), and in response to phytohormones, metal stress, DNA damage, and abiotic stresses (Figure 6). This indicates the multifunctional roles of *GhHDT1502* in cotton growth and development, fiber development, and in response to environmental stimuli. However, further investigations are required to systematically elucidate the function of all *HDACs* in cotton fiber development. Together, we propose that developmental and environmental cues regulate the expression of *HDACs*, which in turn remove the acetylation to suppress the gene transcription involved in various cellular and physiological processes of cotton. On the other hand, the expression of *GhHATs* was also significantly modulated under developmental and environmental cues [56], indicating the dynamic levels of histone acetylation. However, further comprehensive studies are required to elucidate the role of histone acetylation in different aspects of cotton plant development. In brief, our study highlighted the implication of *G. hirsutum HDACs* in phytohormones-mediated developmental processes and abiotic stress adaptation. In addition, this study also highlighted the potential role of *G. hirsutum HDACs* in DNA damage and repair in cotton. This study motivates investigation of the biological and cellular functions of each *G. hirsutum HDAC* in allotetraploid cotton, which will eventually lead to a long-term improvement of *Gossypium* fiber quality and abiotic stress tolerance.

## 4. Materials and Methods

### 4.1. Identification of the HDACs Gene Family

The data of three cotton species, *G. raimondii* (JGI, version), *G. arboretum* (BJI, version1.0) and *G. hirsutum* (NAU, version 1.1) were downloaded from the COTTONGEN (http://www.cottongen.org) [9,10,12]. The HDACs protein sequences from *Arabidopsis* and rice were downloaded from Phytozome (http://phytozome.jgi.doe.gov/pz/portal.html) and then used as queries in BLASTP searches [57] against the *G. raimondii*, *G. arboretum* and *G. hirsutum* genomes. Genes with E-values < 1.0 were selected, and redundant sequences were removed by following the previously published method [58]. Furthermore, InterProScan (http://www.ebi.ac.uk/interpro/search/sequence-search) was used to confirm the presence of the HDACs domain [59]. The physicochemical properties were predicted using ExPASy (http://cn.expasy.org/tools).

### 4.2. Chromosome Location and Duplication of HDACs

The loci of *HDACs* were deduced from the gff3-files of the cotton genome. The localization of HDAC genes on the chromosomes was visualized using the program Circos [60]. The duplication events of the HDACs gene and *Ka*/*Ks* were calculated using the previously published method [61]. The T = *Ks*/2λ equation was used to determine the duplication time and deviation of the *HDAC* gene pairs, assuming clock-like rates of (λ) 1.5 × 10^–8^ substitutions per synonymous site per year for cotton [62].

### 4.3. Phylogenetic Analyses

Multiple sequence alignment was performed for the full-length HDAC proteins using Clustal W with standard settings [63]. A neighbor-joining (NJ) phylogenetic tree was constructed using the full-length HDAC sequences from *G. raimondii*, *G. arboretum* and *G. hirsutum* in MEGA 6.0 [64], with *p*-distance and pairwise gap deletion parameters engaged. The bootstrap test was used with 1000 replicates to evaluate the statistical consistency of each node. To confirm the grades from the NJ method, the minimal-evolution method of MEGA 6.0 was utilized with 1000 replicates as well.

### 4.4. Gene Structure, Domain Composition and Cis-Element Analysis

To produce the exon-intron diagram, we used the BLAST method of both the CDS (CoDing Sequence) and genomic sequences for all the genes and displayed them using the online tool Gene Structure Display Server (http://gsds.cbi.pku.edu.cn) [65], and the NJ tree was constructed with MEGA6.0 as explained above. The deduced HDAC protein sequences of three cotton species were submitted to the online Multiple Expectation Maximization for Motif Elicitation (MEME) version 4.11.1 (http://meme-suite.org/tools/meme) [66], as previously described [58]. For the *cis*-element analysis in the promoter regions, the 1-kb upstream sequences were analyzed in the PlantCARE database (http://bioinformatics.psb.ugent.be/webtools/ plantcare/html/) [67].

### 4.5. Transcriptome Data Analysis and Gene Expression Heatmap

The raw data from the RNA-seq of *G. hirsutum* was downloaded from the NCBI Sequence Read Archive (SRA: PRJNA248163) to calculate the expression level. Tophat and cufflinks were utilized for the RNA-seq expression and fragmented per kilobase per million reads values were made uniform for gene expression. Finally, these values for the *GhHDAC* candidates were extracted from the total expression data and a heat map was generated using MeV 4.0 (http://www.tm4.org/) [68].

### 4.6. Plant Materials, Stress Treatments and qRT-PCR

The *G. hirsutum* cultivar “CRI35” was used for the gene expression analysis. All the sampled tissues obtained from cotton plants were grown under field condition with standard cultural practices to determine the expression analysis [58]. When the cotton plants were in full bloom (approximately 90 days after planting), we collected different cotton tissues, including roots, stems, leaves and flower. Cotton fibers were harvested at 0, 5, 10, 15, 20 and 25 days post anthesis (DPA). All of these samples were immediately frozen in liquid nitrogen and then stored at −80 °C. For hormonal stresses, cotton seeds were surface-sterilized and were allowed to germinate on moist paper. After 2 days of germination, the seedlings of same size were selected and exposed to NaCl (200 mM), polyethylene glycol 4000 (PEG 4000) (15%), cold (4 °C), methyl methanesulfonate (MMS) (250 ppm), auxin (naphthaleneacetic acid (NAA)) (10 μM), abscisic acid (ABA) (10 μM), ZnSO_4_ (1 mM), and CdCl_2_ (1 mM) for 24 h. All these samples were quickly frozen in liquid nitrogen and the total RNA was extracted from all cotton samples using the RNAprep Pure Plant kit (TIANGEN, Beijing, China). A total of 2 μg of RNA was used as the template and the first-strand cDNAs were synthesized using the SuperScript III (Invitrogen, Waltham, MA, USA). Quantitative real-time PCR (qRT-PCR) analysis was performed as described previously [69] by using the specific primers for each *GhHDAC* gene (Table S5). Cotton UBQ7 (UniProt accession number: AY189972) was used as an internal reference gene for normalization of the expression, and three biological replicates were performed for each sample. To calculate the relative expression levels, a comparative 2^−ΔΔCT^ method was used [70]. The heat map for the gene expression profiles was generated with MeV 4.0 (http://www.tm4.org/) [68].

### 4.7. Co-Localization of HDACs with Fiber-Related QTLs

To identify the co-localized QTLs and SNPs for fiber-development-related traits, QTLs and their respective linked molecular markers were retrieved from the Cotton Gen website (https://www.cottongen.org). Molecular markers have genetic positions and to get the physical position information, the sequence of each marker was retrieved from Cotton Gen. Furthermore, the sequence of each marker was compared against *G. hirsutum* in BLAST, using the HAU genome in the cottonFDG website (https://cottonfgd.org/blast/). *HDAC* genes co-localized with QTLs were displayed on chromosomes along with the surrounding loci and QTLs by using mapchart software [71]. Genes identified inside the QTL or ≤500 kb away from an SNP were considered an anchored gene in QTL because the cotton LD decay was approximately 0.80 Mb [72].

## Supplementary Materials

Supplementary materials can be found at https://www.mdpi.com/1422-0067/21/1/321/s1.
