# Supplementary material for: Histone Deacetylase (HDAC) Gene Family in Allotetraploid Cotton and Its Diploid Progenitors: In Silico Identification, Molecular Characterization, and Gene Expression Analysis under Multiple Abiotic Stresses, DNA Damage and Phytohormone Treatments
Source: Int J Mol Sci. 2020 Jan 3;21(1):321. doi: 10.3390/ijms21010321 (PMC6981504; doi:10.3390/ijms21010321)
Supplement: Supplementary file 1 [file ijms-21-00321-s001.zip › HDAC- Supplemenraty FigS1-S3- IJMS.docx]

Research article

Muhammad Imran ^1,2^, Sarfraz Shafiq ^1,3,^*, Muhammad Kashif Naeem ^2^, Emilie Widemann ^4^, Muhammad Zeeshan Munir ^5^, Kevin B. Jensen ^6^ and Richard R.-C. Wang ^6,^*

^1^ School of Life Sciences, Tsinghua University, Beijing 100084, China

^2^ State Key Laboratory of Plant Cell and Chromosome Engineering, Institute of Genetics and Developmental Biology, Chinese Academy of Sciences, Beijing, 100101, China

^3^ Department of Environmental Sciences, COMSATS University Islamabad, Abbottabad campus, Pakistan.

^4^ Department of Biology, University of Western Ontario, 1151 Richmond St, London, Ontario, N6A5B8, Canada.

^5^ School of Biological Science and Technology, Beijing Forest University, 35 Qinghua East Road, Haidian District, 100083 Beijing, China

^6^ Forage & Range Research, United States Department of Agriculture, Agricultural Research Service, Logan, Utah 84322, USA

***** Correspondence: (SS) [sarfraz@mail.tsinghua.edu.cn](mailto:sarfraz@mail.tsinghua.edu.cn); (RW) [Richard.wang@usda.gov](mailto:Richard.wang@usda.gov)

**Figure S1:** Sequence and characteristics of conserved motifs identified in *G. hirsutum, G. raimondii,* and *G. arboretum*.


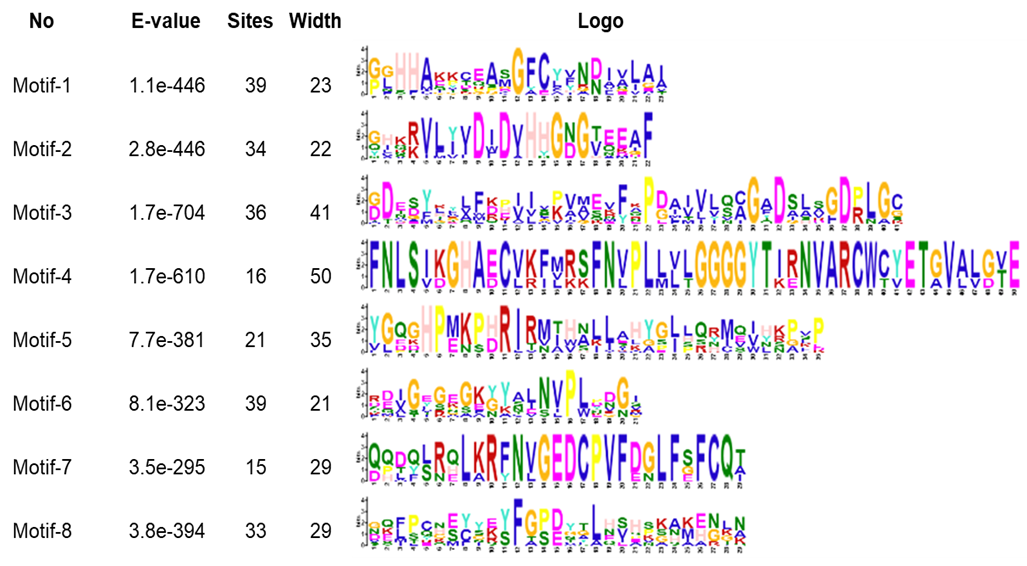


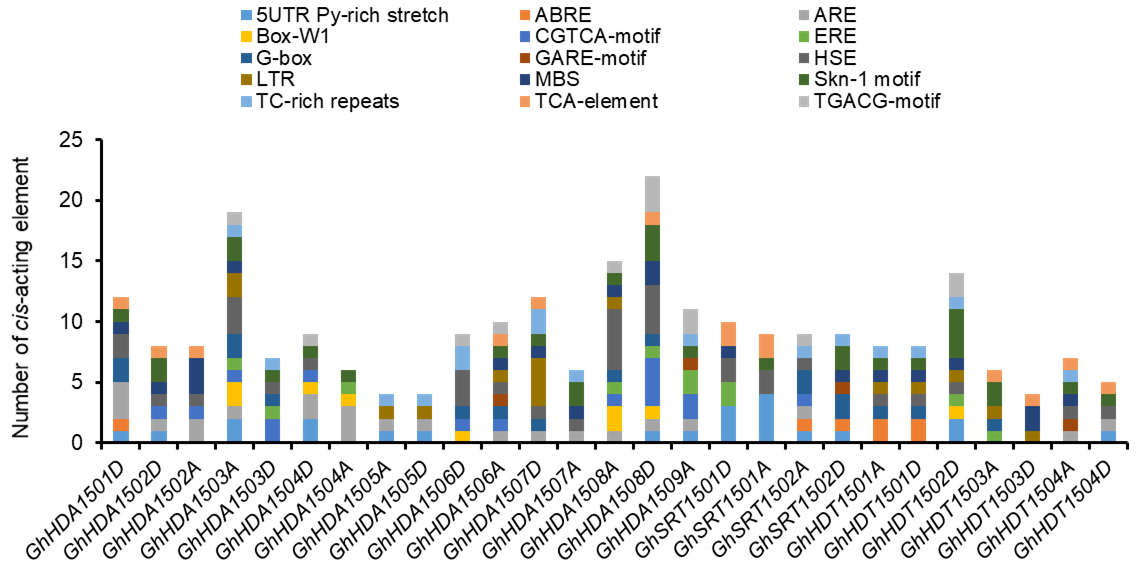


**Figure S2:** Putative *cis*-regulatory elements in the promoter of *G. hirsutum* HDACs.

**Figure S3:** Gene expression pattern of *G. hirsutum* HDACs at different times of cotyledon and root development. The illumine reads of RNA-seq data were retrieved from the NCBI SRA database. The color scale on the bottom of heat map indicates the FPKM-normalized log2 transformed counts.
